# Microbiome and ileum transcriptome revealed the boosting effects of selenium yeast on egg production in aged laying hens

**DOI:** 10.1016/j.aninu.2022.04.001

**Published:** 2022-04-21

**Authors:** Zhexi Liu, Yutao Cao, Yue Ai, Xiaonan Yin, Linli Wang, Mengyao Wang, Bingkun Zhang, Zhengxing Lian, Keliang Wu, Yuming Guo, Hongbing Han

**Affiliations:** a Beijing Key Laboratory of Animal Genetic Improvement, College of Animal Science and Technology, China Agricultural University, Beijing, China; b National Engineering Laboratory for Animal Breeding, College of Animal Science and Technology, China Agricultural University, Beijing, China; c Key Laboratory of Animal Genetics, Breeding and Reproduction of the Ministry of Agriculture and Rural Affairs, College of Animal Science and Technology, China Agricultural University, Beijing, China; d Beijing Alltech Biological Products (China) Co., Ltd., Beijing, China; e State Key Laboratory of Animal Nutrition, College of Animal Science and Technology, China Agricultural University, Beijing, China

**Keywords:** Selenium yeast, Egg production, Gut microbiota, Aged laying hen, Transcriptome

## Abstract

The declines in laying performance during the late production period have adverse effects on the length of the production cycle. Improving the nutrition of laying hens is a crucial measure to reverse this declination. This study investigated the effect of selenium yeast (SY) on egg production, ileal gene expression and microbiota, as well as elucidating their associations in aged laying hens. A total of 375 Jinghong laying hens at 76 weeks old were randomly assigned into 5 dietary treatments, which included a selenium-deficient basal diet based on corn-soybean meal, and dietary supplementation of SY at 0.15, 0.30 and 0.45 mg/kg, and sodium selenite at 0.45 mg/kg. The results showed that SY ameliorated the depression in aged laying performance in the 0.30 mg/kg group (*P* < 0.01). Selenium yeast significantly increased ileum selenium concentration (*P* < 0.05), and SY groups had higher selenium deposition efficiency than the sodium selenite group. Functional enrichment and Short Time-series Expression Miner (STEM) analysis indicated that SY activated metabolic progress (e.g., glycerolipid metabolism, glycerophospholipid metabolism, and fatty acid metabolism), immune response and oxidative stress response. Four hub genes including thioredoxin reductase 1 (*TXNRD1*), dihydrolipoamide dehydrogenase (*DLD*), integrin linked kinase (*ILK*) and leucine zipper tumor suppressor 2 (*LZTS2*) were involved in intestinal metabolism which was closely associated with selenium deposition/status. Moreover, the relative abundance of *Veillonella*, *Turicibacter* and *Lactobacillus* was significantly increased, but the relative abundance of Stenotrophomonas was significantly decreased by SY supplementation. Multi-omics data integration and Canonical correspondence analysis (CCA) showed that both the ileum selenium content and the laying rate were highly correlated with pathways and bacteria enriched in metabolism and immune response. Meanwhile, the “switched on” gene prostate stem cell antigen (*PSCA*) had a positive relationship with *Veillonella* and a negative relationship with the opportunistic pathogens *Stenotrophomonas*. Overall, our study offered insight for the further exploration of the role of SY on boosting egg production and balancing ileum intestinal flora in aged laying hens.

## 1 Introduction

With the improvement of production performance in laying hens, the laying age of commercial laying hens extended from the original 72 to 80 weeks (Schultedrüggelte and Thiele, 2013). Hence, there has been an explosion of interest in keeping hens in production for longer periods. In general, the main reason for replacing flocks around 72 weeks of age is the decline in egg production with an impaired eggshell quality (Bain et al., 2016). Thus, it should be concerned about laying persistency and the stability of egg quality in a longer laying cycle.

Selenium (Se), as a food additive and micronutrient, plays essential roles in laying performance, protecting against intestinal inflammation, and modulating microbiota in hens (Short et al., 2018). A large number of studies showed that selenium yeast (SY) supplementation enhanced egg production in laying hens (Liu et al., 2020). Now, there is rising attention on the effects of Se on the gut. In general, diet and feed additives are the most common factors that can influence the gastrointestinal microbiota diversity, composition, and structure to maintain intestinal homeostasis (Stanley et al., 2013). The health of intestine and the balance of intestinal microbiota may be controlled by the effect of Se on relieving oxidative stress. Moreover, the chemical form and different doses of Se should be considered as a dietary nutritional supplement used to improve gut health (Zhai et al., 2019). Variations in Se intake have multifaceted effects including optimizing the gut microbiota to avoid intestinal dysfunctions (Zhai et al., 2018). The benefits of different chemical forms of Se are dominated in diverse functions, and organic forms of Se, such as SY, are considered more efficient than inorganic forms. The links between changes in gut microbiota and selenoprotein gene expression after Se supplementation in mice have been illustrated in early findings, dietary Se altered the composition of the gut microbiota, and also influenced both the host Se state and selenoprotein expression (Zhai et al., 2018). An amount of gut microbiota could synthesize selenoproteins and essential Se to ensure optimal growth and some metabolic functions (Hrdina et al., 2009; Kasaikina et al., 2011). Under Se-limiting conditions, the gut microbiota might compete with the host for the limited Se supply (Hrdina et al., 2009). Therefore, it is essential to supplement selenium to adequate status to maintain intestine health and the balance of the microbiota.

The pattern of host–microbe interactions drives the genetic and phenotypic diversities of gut microbiota to affect the physiological, immunological, and nutritional status of the host (Kreznar et al., 2017). Recent studies demonstrated that the specific microbes in the gut, such as *Collinsella*, played an essential role in the regulation of human complex traits via modulating the expressions of host genes (Richards et al., 2019); indicating that there was a reciprocal regulation between the host and the gut microbiota. However, the potential effect of SY on egg production in aged laying hens is still ambiguous, and the associations between dietary SY, laying performance, microbiota, and the host's gene expression require further elucidation. To clarify how dietary SY affected the intestinal tract in aged laying hens, transcriptomic and bioinformatics analysis was performed to explore the relationships among laying performance, SY intakes, and gene expression of the host. Additionally, 16S rRNA sequencing was used to analyze the composition of gut microbiota in aged laying hens maintained on selenium-deficient (SD), sodium-selenite (SS), and selenium-yeast (SY) diets. Moreover, multi-omics correlation analysis revealed the associations between changes of pathways and microbiota affected by Se supplementation with the laying performance in aged laying hens.

## 2 Materials and methods

### 2.1 Animal ethics

The experimental animal protocols for this study were approved by the Animal Care and Use Committee of China Agricultural University (No. AW05060202–1).

### 2.2 Animals and treatments

During the Se-consumption period, a total of 375 Jing Hong laying hens at 76 weeks of age were fed with a basal corn-soybean diet for 6 weeks (from 76 to 82 weeks of age). The basal diet had an average basal Se content of 0.056 mg/kg, to consume the body's stores of Se and generate the Se-deficient hens. The composition and nutrient level of the basal diet are shown in Table 1, the doses of Se were premixed in diets, and the corn-soybean diet was formulated to satisfy the Chinese Feeding Standard of Chickens (NY/T33-2004). After Se-consumption, Jing Hong laying hens with similar laying rate were randomly allocated to 5 treatment groups with 5 replicates (15 chickens per replicate) each. One group was fed the basal diet (SD), the remaining 4 groups were supplemented with SY at 0.15, 0.30, and 0.45 mg/kg (Alltech, Nicholasville, KY, USA) namely SY0.15, SY0.30 and SY0.45 group, and sodium selenite at 0.45 mg/kg (SS0.45 group) for 12 weeks (from 83 to 95 weeks of age). The Se contents of different diets are shown in Table 2. Batches of the experimental diets were produced every 4 weeks to prevent the feed from mildewing. The hens were housed in an environmentally controlled room maintained at 25 °C and had a daily lighting schedule of 16 h of light (from 05:00 to 21:00) and 8 h of darkness (from 21:00 to 05:00).

**Table 1:** Experimental diet composition and nutrients (as-fed basis, %).

Diet composition	Content	Nutrient level	Content
Corn	60.50	AME, MJ/kg	2.57
Soybean meal	21.60	Crude protein	15.0
Wheat	4.10	Lysine	0.74
Cottonseed meal	2.00	Methionine	0.30
Soybean oil	0.50	Methionine ＋ Cysteine	0.55
Calcium carbonate (GR)	9.50	Calcium	3.70
Calcium phosphate (GR)	1.00	Available phosphorus	0.31
DL-Methionine	0.08	Total phosphorus	0.54
Phytases	0.015	Selenium^3^, mg/kg	0.056
Vitamin premix^1^	0.035		
Mineral premix^2^	0.15		
Sodium chloride (GR)	0.30		
50% Choline chloride	0.10		
Experimental additives	0.12		
Total	100.00		

GR = guaranteed reagent.

1 Provided per kilogram of diet: vitamin A, 12,500 IU; vitamin D_3_, 2,500 IU; vitamin E, 18.75 mg; vitamin K_3_, 2.65 mg; vitamin B_1_, 2 mg; vitamin B_2_, 6 mg; vitamin B_12_, 0.025 mg; biotin, 0.325 mg; folic acid, 1.25 mg; niacin, 50 mg.

2 Provided per kilogram of diet: Cu, 8 mg; Fe, 80 mg; Zn, 80 mg; Mn, 60 mg; I, 1.2 mg. Selenium in each treatment group is shown in the experiment design in Table 2.

3 This value is measured.

**Table 2:** Experiment design (supplemented dose, mg/kg).^1^

Item	Control	Sodium selenite	Selenium yeast		
	SD	SS0.45	SY0.15	SY0.30	SY0.45
Measured values^2^	0.056 ± 0.012	0.480 ± 0.016	0.211 ± 0.015	0.377 ± 0.049	0.552 ± 0.030

SD = selenium deficient; SS = sodium selenite; SY = selenium yeast.

1 The treatment groups SD, SS0.45, SY0.15, SY0.30 and SY0.45 represented the basal Se-deficient diet, 0.45 mg/kg sodium selenite added to the basal diet, 0.15, 0.30 and 0.45 mg/kg selenium yeast added to the basal diet, respectively.

2 The measured values represented by mean ± SEM.

### 2.3 Data and sample collection

Blood samples (8 mL per laying hen) were taken from the main wing vein and collected into an anticoagulant tube every 2 weeks during the Se-consumption period. Plasma was separated by centrifugation at 4 °C, 1,400 × *g* for 10 min and stored at −30 °C for further analysis. After Se supplementation for 12 weeks, 10 randomly chosen laying hens from each dietary treatment were slaughtered, the ileum and its chyme were sampled and frozen in liquid nitrogen immediately. All samples were stored at −80 °C prior to analysis.

### 2.4 Production performance

The laying rate of aged laying hens was measured from 83 to 95 weeks of age. Daily egg production was determined per replicate unit at 14:30. The monthly laying rate was measured by daily egg production in 4 weeks.

### 2.5 Determination of Se content

To determine the Se content in feed, plasma, and ileum. All samples (0.5 to 1 g for feed and ileum, and 0.5 to 1 mL for plasma) were digested in a mixture of concentrated nitric acid and perchloric (2 HNO_3_:1 HClO_4_) for about 2 h at 200 °C until white fumes appeared. Then 5 mL hydrochloric acid (1 HCl:4H_2_O) solution was added in the mixture and heated until white fumes appeared. After the mixture cooled, 20 mL ethylenediaminetetraacetic acid (EDTA) was added in the digested sample and pH was adjusted to 1.5–2.0. After which, 3 mL 2,3 DiAminoNaphthalene (DAN) was added in the mixture and heated in the boiled water for 5 min. After the mixture cooled, 4 mL cyclohexane was added and the mixture was shaken for 8 min. The supernatant was measured by fluorescence method using Hitachi 850 fluorescence spectrophotometer (Tokyo, Japan) (Liao et al., 2012).

### 2.6 Measurement of β-galactosidase assay and β-galactosidase staining

In vitro β-galactosidase measurement was performed on ileum of aged laying hens by β-Galactosidase Enzyme Assay System with Reporter Lysis Buffer (E2000, Promega, WI, USA). Lysates were prepared using 1 × Reporter Lysis Buffer and then Assay 2 × Buffer and β-galactosidase assay stop solution to develop the reaction. For β-galactosidase staining, cryosections (8 μm) from ileum frozen in OCT were stained with β-Galactosidase Staining Kit (9860, Cell Signaling Technology, BSN, MA, USA). Frozen tissues were fixed by 1 × fixative solution for 15 min and stained by a prepared β-galactosidase staining solution and incubated at 37 °C overnight. The entire images were captured by APERIO CS2 (Leica, Germany).

### 2.7 RNA extraction and RNA-sequencing

Total RNA was isolated from the ileum using RNA Isolated Kit (RN4402, Aidlab Biotechnologies, Beijing, China) according to the manufacturer's protocol. Quality and quantity measurements of the extracted RNA were performed using NanoPhotometer (IMPLEN, CA, USA) and a Qubit Fluorometer (Life Technologies, CA, USA), respectively. RNA Integrity Numbers (RIN) were determined using 2100 RNA Nano 6000 Assay Kit (Agilent Technologies, CA, USA). Paired-end transcriptome sequencing was performed using an Illumina Novaseq6000 sequencing platform at Annoroad Biotechnology Co. Inc. (Beijing, China).

### 2.8 Transcriptome bioinformatic and statistical analysis

After the quality raw RNA-seq data was controlled by removing low-quality reads and repeated sequences by fastp Version 0.20.0, clean data was obtained for further analysis. The datasets generated and analyzed in the current study are available in the Sequence Read Archive (SRA) database at NCBI under BioProject ID PRJNA664535. Read count matrices were obtained using the FeatureCounts package, the data was aligned to reference chicken genome (GRCg6a) downloaded from Ensembl (https://asia.ensembl.org/index.html) performed by HISAT2. Differential expressed genes (DEG) were accessed using the R package DESeq2. The significance thresholds were FDR <0.05 and |log2foldchange| > 1.0, and heatmaps were drew using R package ComplexHeatmap. Functional enrichment analysis was performed by PANTHER (http://pantherdb.org/) and KOBAS (http://kobas.cbi.pku.edu.cn/kobas3/genelist/) websites, for Gene Ontology (GO) and Kyoto Encyclopedia of Genes and Genomes (KEGG) analysis, respectively. The Top 80 significant pathways (*P* < 0.05) were selected for further visualization and were performed with R package ggplot2.

The expression data of ileum tissues in the SD, SY0.15, SY0.30, and SY0.45 groups were subjected to weighted gene co-expression network construction using the weighted gene co-expression network analysis (WGCNA) package and following the pipeline. To identify modules that were significantly associated with the Se content in the ileum, Pearson correlations (*P* < 0.05 and |*r*| > 0.4) were carried out. The candidate hub genes with high intramodular connectivity in modules were explored through the gene significance (GS > 0.7) and the module membership (MM > 0.9) which was associated with Se deposition. Ultimately, the hub genes were top 30 in each module chosen by soft Connectivity function. The gene–gene interaction network was constructed and visualized by Cytoscape software, and DEG in any SY supplementation groups were marked as V if founded in modules.

To investigate the dynamic changes in gene expression supplemented by different doses of SY, Short Time-series Expression Miner (STEM) (Ernst and Bar-Joseph, 2006) was performed. DEG among SY groups were stratified along with supplementation gradients and log transformation. We expected to capture nicety tendencies as well as a high consistency within clusters. Therefore, Maximum profiles were set relatively high at 100 and any cluster required intra-profiles correlation that were higher than 0.88. Then gene set enrichment analysis (GSEA) was performed to get more functional information in the clusters of interest. Single-sample GSEA (ssGSEA) algorithm was used combined with other results for further analysis and was implemented in R package GSVA. A matrix of enrichment scores for each gene set and the sample was obtained, with DESeq2 being normalized results as input data. When it came to visualization, the only results with standard deviation among samples were no more than 0.1.

Next, the switched on or off (ON and OFF) genes regulated by SY were screened. First, genes whose variance was more than 1.5-fold of quartile deviation were filtered out. Then, if the median of a gene's expression within a given group equals zero, the genes were defined as OFF-genes, making ON-genes were an opposite state. Meanwhile, a standard deviation of genes was no more than 1.0 counts and the mean of gene expression and each gene should show a statistical significance (*P* < 0.05) through Wilcoxon signed-rank test. Two states of the control group were respectively intersected with the opposite states of treatments. Then genes that switched between ON and OFF states were derived. The Protein–Protein Interaction (PPI) was then performed on the STRING (https://string-db.org/) website to exhibit the relationship between ON and OFF genes. Only the connected nodes with confidence more than 0.4 were displaced. Moreover, the changes of redox-related and aging-related genes were investigated to get more insights about Se yeast functions, the gene sets were retrieved on the Molecular Signatures Database website (https://www.gsea-msigdb.org/gsea/msigdb/genesets.jsp) (Table S1).

### 2.9 16S rRNA microbial community analysis

The ileum chyme genome was extracted using a FastDNA™ SPIN Kit (MP Biomedicals, Santa Ana, CA, USA). The 16S rRNA gene comprising V3–V4 regions was amplified as described previously (Munyaka et al., 2015). Deep sequencing was performed on Miseq platform at Allwegene Company (Beijing, China). The datasets generated and analyzed in the current study are available in the Sequence Read Archive (SRA) database at NCBI under BioProject ID PRJNA664532.

After the run, image analysis, base calling and error estimation were performed using Illumina Analysis Pipeline Version 2.6. The raw data were first screened and sequences shorter than 230 bp, had a low quality score (≤20), contained ambiguous bases or did not exactly match to primer sequences and barcode tags, were removed from consideration. Qualified reads were separated using the sample-specific barcode sequences and trimmed with Illumina Analysis Pipeline Version 2.6, then the dataset was analyzed using QIIME. All sequences were used for the comparison of relative abundance of bacterial taxa and were clustered into operational taxonomic units (OTUs) according to a 97% similarity, to generate rarefaction curves and to calculate the richness and the diversity indices. The Ribosomal Database Project (RDP) Classifier tool was used to classify all sequences into different taxonomic groups. Finally, the relative abundance of each bacterial taxa was analyzed by QIIME and predicted functional genes were analyzed by Phylogenetic Investigation of Communities by Reconstruction of Unobserved States (PICRUSt) based on the KEGG pathway (Langille et al., 2013).

### 2.10 Host transcriptome–microbiota correlation analysis

The associations between OTUs and phenotypes (laying rate and Se content in ileum) were explored using built-in function cor of the R package. Correlation between GSVA output and OTUs was performed using the dmic for similarity measurement implemented in Hierarchical All-against All (HAllA) software developed for multi-omic data sets, which referred to this study (Lavelle et al., 2019). Significantly correlated pairs were retained with default parameters. Further visualization was performed using the R package ComplexHeatmap. The associations between ON/OFF and selenoprotein genes and ileum bacteria OTUs were explored by Pearson correlation, R package Hmisc was used for calculating the Pearson correlation and the asymptotic *P*-values between 2 high-dimensional data sets, and the significant standards were set as *P* < 0.05, |*r*| > 0.6. Further visualization was performed using R packages ComplexHeatmap as well as ggplot2. Lastly, CCA was performed using the R package vegan. Along with 2 phenotype traits, the Se content in the ileum and laying rate, GSEA results for significant clusters in STEM were used as explanatory variables with the collinearity removed.

### 2.11 Quantitative real-time PCR(qRT-PCR) analysis

Expression of mRNA from the transcriptomic analysis was verified by qRT-PCR with cDNA from 12 ileum tissue samples. Primer Primer (Version 5.0) with default parameters was used to design primers for selected genes (Table 3). *Beta-actin* gene served as a housekeeping gene. qRT-PCR analysis was conducted with a reaction volume of 20 μL containing 10 μL Premix (FP209-2, Tiangen, Beijing, China), 0.6 μL forward and reverse primers, 1 μL cDNA, and 7.8 μL DNase/RNase-Free Deionized Water. The reaction conditions followed the protocols and instructions. The 2^−ΔΔCT^ method was used to quantify the relative changes in gene expression versus those of *β-actin* from qRT-PCR experiments.

**Table 3:** Primers used for qRT-PCR.

Gene	Sequence (5′-3′)	Product length, bp	Annealing temperature, °C
*OVA*	F: AAGCAGGCAGAGAGGTGGTAGG R: ACGGCGTTGGTTGCGATGTG	121	62
*TXNRD1*	F: TGAACTGGGGCTATCGGGTA R: TTTTCCCCGGACAGTAAGGC	243	56
*PSCA*	F: GGCTGTGAGGCATCATGTCAAG R: TGTTCAGGAAGGTCCAGAGCAG	169	56
*β-actin*	F: GAGAAATTGTGCGTGACATCA R: CCTGAACCTCTCATTGCCA	152	56

*OVA* = ovalbumin; *TXNRD1* = thioredoxin reductase 1; *PSCA* = prostate stem cell antigen.

### 2.12 Statistical analysis

Results were presented as mean ± SEM, and differences between 2 groups were analyzed by an unpaired Student's *t*-test, data that involved more than 2 groups was analyzed with one-way ANOVA, along with the Duncan test (SPSS for Windows, Version 25; IBM). Statistical differences were considered significant at *P* < 0.05. Phenotypic data presentation was carried out using GraphPad Prism (Version 7.0, GraphPad Software Inc, San Diego, CA, USA). Each experiment was repeated at least 3 times.

## 3 Results

### 3.1 A proper dose of selenium yeast supplementation ameliorates the depression in laying performance during the late laying period

**Fig. 1:**
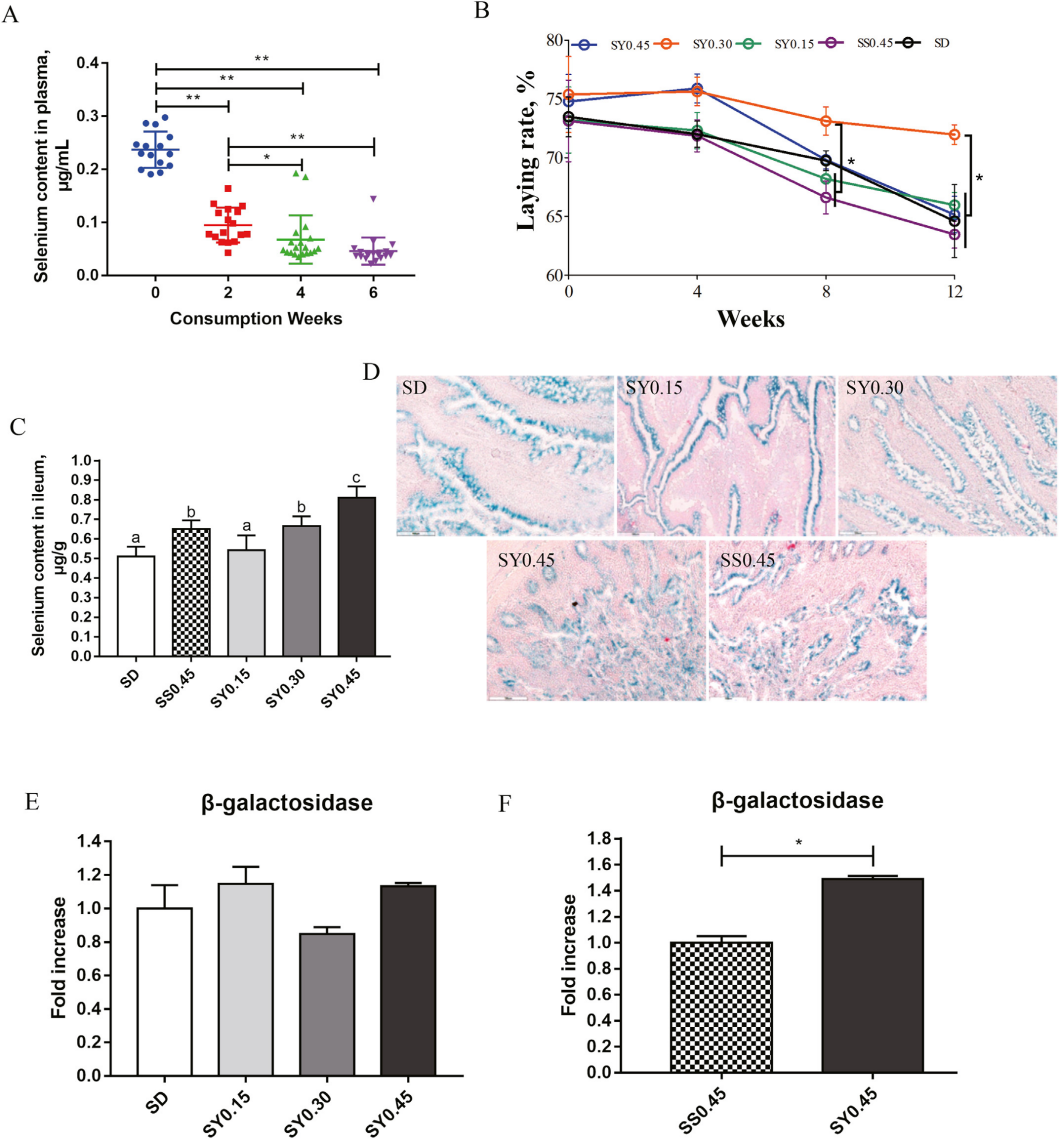
The effects of dietary selenium yeast (SY) supplementation on aged laying hens. (A) Selenium content in plasma during the consumption period, each color means different week in consumption period. (B) The laying rate during the supplementation period. (C) Selenium status in ileum after selenium supplementation. Data was analyzed with one-way ANOVA, along with Duncan test. ^a, b, c^ Different letter means there was a significant difference between groups (*P* < 0.05). (D) β-galactosidase staining images from ileum tissue (scale bar, 100 μm). (E) The level of β-galactosidase activity in the ileum was measured between the selenium-deficient (SD) group and each SY group by unpaired Student's *t*-test. (F) The level of β-galactosidase activity in ileum between the SS0.45 group and SY0.45 group. Data are represented as mean ± SEM. ∗ means *P* < 0.05 and ∗∗ means *P* < 0.01. SS = sodium selenite.

Following selenium-deficient treatment, the Se content in plasma dropped dramatically (*P* < 0.01) and then remained at a lower status (Fig. 1A), suggesting that the Se in aged laying hens was depleted. During the Se supplementation period, laying performance exhibited a gradual decline trend. Within 4 weeks, no significant differences in laying performance were found. However, after 8 weeks of supplementation, laying performance was significantly higher (*P* < 0.01) in the SY0.30 group than the SS0.45 group and SY0.15 (Fig. 1B and Table 4). The polynomial contrast results showed that the response of Se-yeast to egg production tends to be linear. Moreover, the Se content in the high dose group dramatically increased compared to the SD group. Meanwhile, the Se content in the SY0.45 group was higher than the SS0.45 group at the same dose (Fig. 1C).

**Table 4:** The egg production of aged laying hens in selenium supplementation period (%).

Treatment	4 weeks	8 weeks	12 weeks
SD	71.99 ± 1.12	69.74 ± 0.85^ab^	63.52 ± 3.14^b^
SY0.15	72.32 ± 1.55	68.20 ± 1.25^b^	65.97 ± 1.08^b^
SY0.30	75.64 ± 1.22	73.11 ± 1.20^a^	71.96 ± 0.83^a^
SY0.45	75.91 ± 1.24	69.62 ± 0.78^ab^	65.16 ± 1.55^b^
SS0.45	71.88 ± 1.36	66.62 ± 1.39^b^	63.47 ± 1.16^b^
SY-Linear	0.021	0.324	0.060
SY-Quadratic	0.261	0.588	0.159
*P*-value	0.051	0.001	<0.001

SD = selenium deficient; SS = sodium selenite; SY = selenium yeast.

^a, b^ Values in the same row with different superscripts are significantly different (*P* < 0.05) by one-way ANOVA.

In general, the peaking production of commercial laying hens usually remains at the age of 60-weeks, and after that period the laying rate and egg quality begin to decline. Thus, age may be one of the leading factors causing egg performance reduction. Additionally, the effect of Se supplementation on ileum aging was investigated by testing β-galactosidase activity. There were no significant differences between the SD group and the SY groups (Fig. 1D, E). Interestingly, a significant difference was observed between the SS group and the SY group (*P* < 0.05) (Fig. 1F).

### 3.2 Transcriptome analysis revealed selenium yeast supplementation may affect metabolic pathways and immune response in the ileum in aged laying hens

**Fig. 2:**
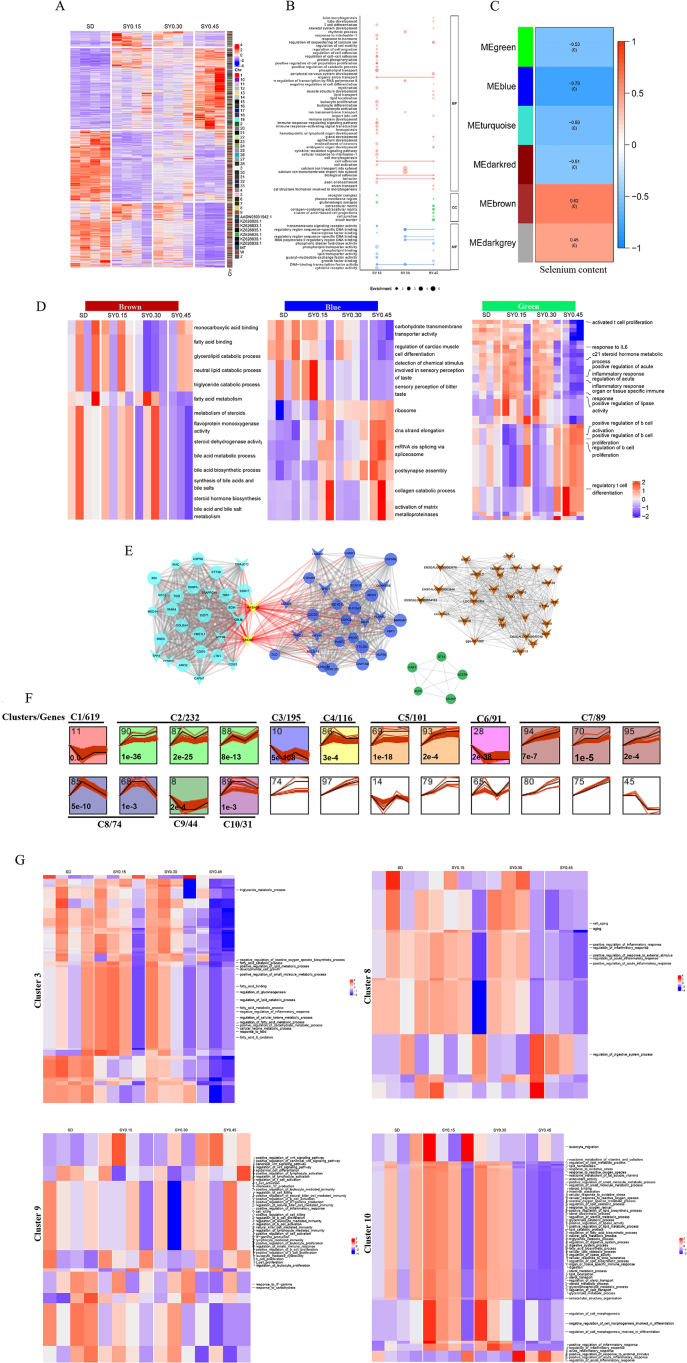
Transcriptomic analysis revealed differential and dynamic effects of selenium yeast (SY) on ileal gene expression. (A) The heatmap of DEG between the selenium-deficient (SD) group and SY groups, where the red means up-regulated genes, and the blue means down-regulated genes, and the right bands mean the location of genes on the chromosome. (B) Enriched GO categories for DEG were identified among 3 SY groups compared with the SD group. (C) Relationships between modules and selenium content in the ileum. Each band of the matrix contains the corresponding correlation between gene module and selenium content in the first line and *P*-value in the second line. The intensity and direction of correlations are indicated on the right side of the heatmap (red, positively correlated; blue, negatively correlated). (D) Functional enrichment analysis of genes in interesting modules by gene set enrichment analysis (GSEA). (E) The interactive network plot of hub genes identified within the blue, green, brown and turquoise modules. The node size and edge number are proportional to the degree and connection strength, respectively. Genes with high connection strength were colored by yellow, and the red line means hub genes have a direct connection with high connection strength genes. The genes with a V-shape represent the differentially expressed hub genes. (F) Identification of 10 significant gene cluster profiles by Short Time-series Expression Miner (STEM). Colored block trend: significant enrichment trend (*P* < 0.05). The number of genes in each significant cluster was shown after the cluster number. (G) Functional enrichment analysis of genes in interesting clusters by GSEA.

To figure out the effects of different doses and forms of Se, the gene expression profile was analyzed using RNA-seq. Compared with the SD group, 1,857 (538 upregulated; 1,319 downregulated), 627 (207 upregulated; 420 downregulated) and 3,305 (1,379 upregulated; 1,926 downregulated) differentially expressed genes (DEG) were obtained in the SY0.15 group, SY0.30 group and SY0.45 group, respectively (Fig. 2A and Table S2). Simultaneously, 366 DEG were shared among 3 groups. The functional enrichment results based on GO and KEGG pathway analysis showed that the common enriched pathways in 3 groups were Glycerophospholipid metabolism, Glycerolipid metabolism and DNA-binding transcription factor activity (Fig. 2B, Fig. S1A and Table S3). Due to 0.30 mg/kg SY alleviating the depression in laying performance, the unique pathways in the SY0.30 group were focused on, including the ion transmembrane transport, calcium ion transport into the cytosol, and MAPK signaling pathway (Fig. 2B and Fig. S1A).

The relationship between Se content and gene expression profile in the ileum was analyzed by the weighted gene co-expression network analysis (WGCNA). WGCNA analysis showed that all the expressed genes were segmented into 17 modules (Fig. 2C, Fig. S1B and Table S3). The genes in the MEbrown and MEdarkgrey module were positively correlated with the ileum Se content, but they were negatively correlated in MEgreen, MEblue, MEturquoise, and MEdarkred. GSEA analysis results showed that genes in these modules were major enriched in fatty acid metabolism, immune response and carbohydrate transmembrane transporter activity (Fig. 2D). Some hub genes were identified from these modules (Fig. 2E and Fig. S1C), including *TXNRD1* (selenocompound metabolism pathway), *DLD* (citrate cycle/TCA cycle pathway and pyruvate metabolism pathway), *ILK* (regulation of canonical Wnt signaling pathway) (Fig. S1D and E). Furthermore, STEM analysis results showed that total DEG were clustered into 10 profiles (Fig. 2F). The biological functions of 4 interested clusters (clusters 3, 8, 9, 10) were enriched in metabolic process, intestinal absorption and digestion, aging, inflammatory response, reactive oxygen species biosynthetic process and developmental cell growth (Fig. 2G and Table S3).

Generally, SY is a high-quality organic Se source for animals that is incorporated into proteins structures to improve bioavailability. Differences in the sources of Se may have diverse effects on the gene expression of hosts. Thus, the effect of different Se sources under the adequate Se status was investigated in aged laying hens. A total of 586 DEG were obtained in the SY0.45 group relative to the SS0.45 group (Fig. S1F and Table S2). GO enrichment analysis indicated that the DEG were enriched in fatty acid metabolic process, cellular lipid metabolic process, and oxidoreductase activity (Fig. S1G and Table S3). Meanwhile, KEGG analysis suggested that the major differences were in Glycerophospholipid metabolism, Glycerolipid metabolism, PPAR signaling pathway, mTOR signaling pathway, and Fatty acid degradation (Fig. S1H and Table S3).

To validate the transcriptomic analysis results, the relative gene expression of the candidate gene (*OVA*, *TXNRD1*, *PSCA*) was performed by quantitative real-time PCR (qRT-PCR). The qRT-PCR results showed a similar expression pattern compared with RNA-seq results, indicating that these genes were validated (Fig. S1I).

### 3.3 Selenium yeast supplementation may affect the ileal metabolic process by changing the expression of redox and aging genes

**Fig. 3:**
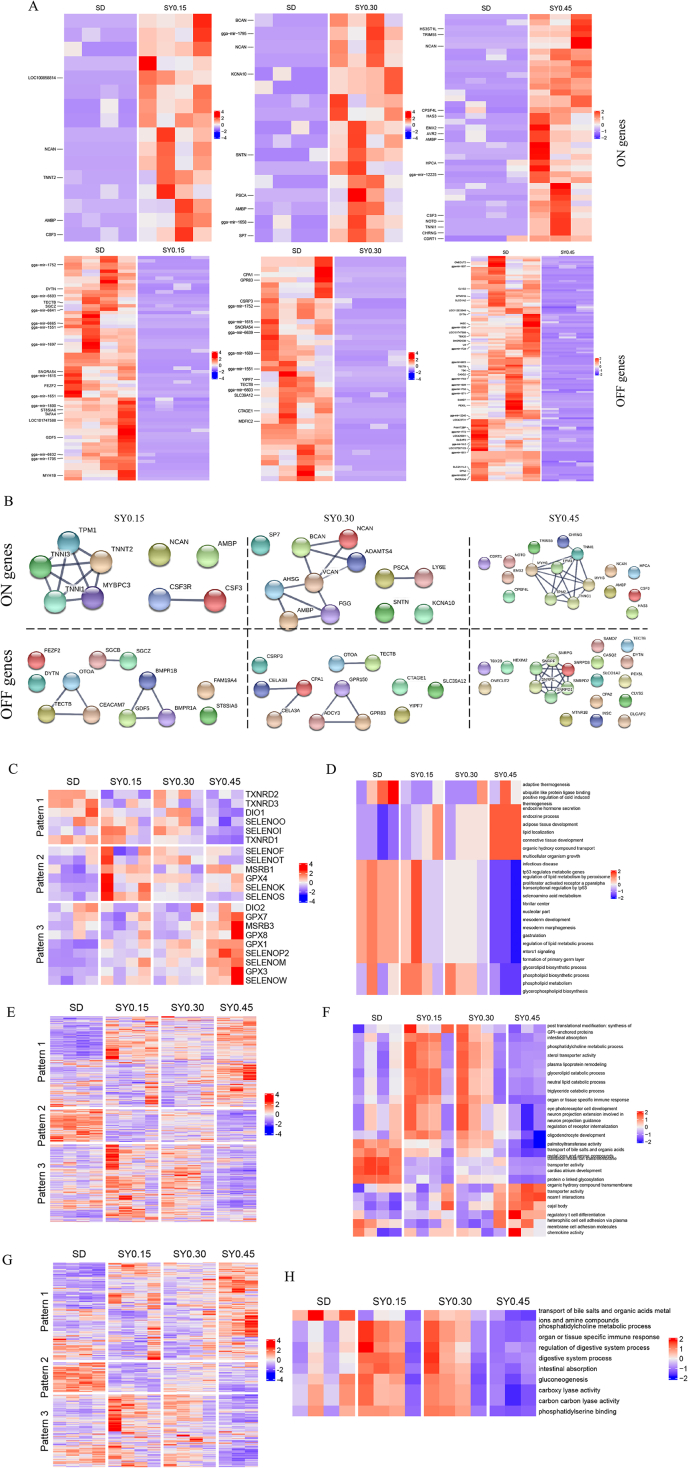
Functional enrichment analysis of genes associated with redox and aging after selenium yeast (SY) supplementation. (A) Hierarchical clustering of identified switched ON and OFF genes in different doses of SY. (B) Protein–protein interaction (PPI) network plot of ON and OFF genes in respective doses. (C) Hierarchical clustering of selenoprotein genes in different doses of selenium yeast (SY). (D) Functional enrichment analysis of selenoprotein genes in different doses of SY. (E) Hierarchical clustering of redox-associated genes in different doses of SY. (F) Functional enrichment analysis of redox-associated genes in different doses of SY. (G) Hierarchical clustering of aging-associated genes in different doses of SY. (H) Functional enrichment analysis of aging-associated genes in different doses of SY.

The gut could turn ‘ON’ or ‘OFF’ collections of the genes via epigenetics and receptor-driven transcription factors in response to the perceived environment (Gillespie et al., 2019). Thus, the genes switched-on or off under different doses of SY supplementation were detected. Twenty-seven ON genes, including; prostate stem cell antigen (*PSCA*), neurocan (*NCAN*) and brevican (*BCAN*), and 64 OFF genes, including; ST8 alpha-N-acetyl-neuraminide alpha-2,8-sialyltransferase 6 (*ST8SIA6*), cysteine and glycine rich protein 3 (*CSRP3*) were detected in different SY groups (Fig. 3A and Table S1). Furthermore, PPI analysis showed that switched-on genes including *NCAN*, *BCAN*, and colony stimulating factor 3 (*CSF3*) were associated with carbohydrate metabolism or immune response. Switched-off genes including *ST8SIA6*, solute carrier organic anion transporter family member 1A2 (*SLCO1A2*) were associated with carbohydrate biosynthesis and lipid metabolism, respectively (Fig. 3B).

Dietary intake of Se affects the hierarchical pattern of organ-specific selenoprotein expression. Different doses of SY supplementation can determine tissue Se deposition. Thus, the patterns of selenoprotein gene expression in the ileum were examined. Twenty-one selenoprotein genes were divided into 3 expression patterns. The genes of pattern 1, including thioredoxin reductase 1 (*TXNRD1*), thioredoxin reductase 2 (*TXNRD2*), thioredoxin reductase 3 (*TXNRD3*), iodothyronine deiodinase 1 (*DIO1*), selenoprotein O (*SELENOO*) and selenoprotein I (*SELENOI*), decreased in groups with Se supplementation. Six selenoprotein genes, glutathione peroxidase 4 (*GPX4*), methionine sulfoxide reductase B1 (*MSRB1*), selenoprotein F (*SELENOF*), selenoprotein T (*SELENOT*), selenoprotein K (*SELENOK*), selenoprotein S (*SELENOS*) in pattern 2 exhibited the highest expression in the SY0.15 group and decreased with the dose increase. The expression of 9 selenoproteins in pattern 3, namely iodothyronine deiodinase 2 (*DIO2*), glutathione peroxidase 1 (*GPX1*), glutathione peroxidase 3 (*GPX3*), glutathione peroxidase 7 (*GPX7*), glutathione peroxidase 8 (*GPX8*), methionine sulfoxide reductase B3 (*MSRB3*), selenoprotein P2 (*SELENOP2*), selenoprotein M (*SELENOM*), selenoprotein W (*SELENOW*), were increased with dose increasing of Se (Fig. 3C). The GSEA analysis showed that the effects of SY supplementation were associated with the expression of selenoproteins enriched in adipose tissue development, lipid localization, selenoamino acid metabolism and Glycerophospholipid biosynthesis (Fig. 3D and Table S3).

Reactive oxygen species (ROS) production and antioxidants Se play important roles in the regulation of redox processes. Thus, the expression of genes associated with the redox process had to be evaluated (Table S1). The expression of these genes was enriched in intestinal absorption, glycerolipid catabolic process, transport of bile salts and organic acids, and regulatory T cell differentiation (Fig. 3E, F and Table S3). To explore the effect of Se intake on aging, the expression of genes associated with aging was analyzed (Fig. 3G and Table S1). The functions of these genes were mainly enriched in intestinal absorption, digestive system process, organ or tissue-specific immune response, and gluconeogenesis (Fig. 3H and Table S3).

### 3.4 Se supplementation modulated the composition and structure of gut microbiota in aged laying hens

**Fig. 4:**
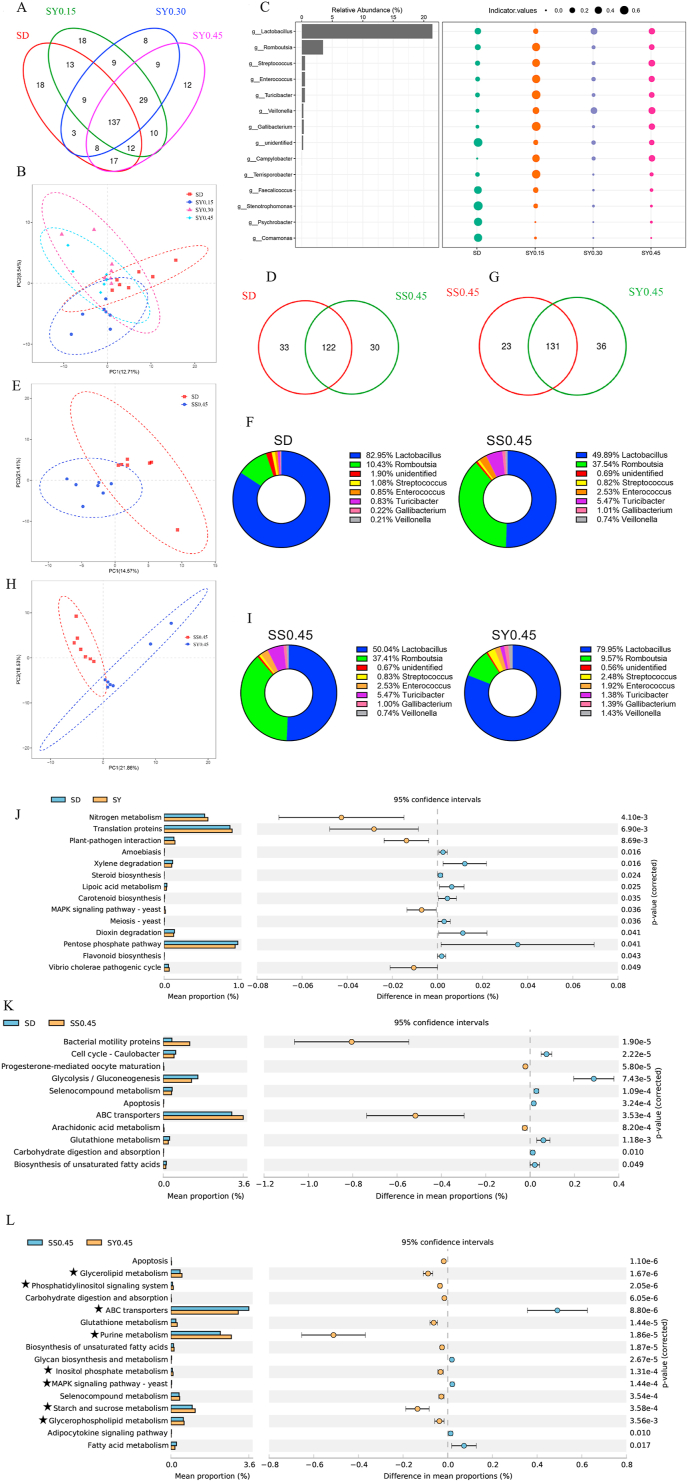
The effects of selenium yeast (SY) supplementation on the composition and function of gut microbiota. (A) The Venn diagram shows the common and unique operational taxonomic units (OTUs) in ileum samples in SD and SY groups. (B) The Partial Least Squares Discrimination Analysis (PLS-DA) plot between the SD and SY groups. (C) Indicator plot of relative abundance of the genera in the ileum by SY supplementation, bubble size represents the indicator in each group, and the larger bubble means larger indicative of the species in this group. (D) Venn diagram shows the common and unique OTUs in ileum samples in SD and SS0.45 groups. (E) PLS-DA plot of ileum microbiota between the SD and SS0.45 groups. (F) Relative abundance of the genera detected in the ileum in SD and SS0.45 groups. (G) Venn diagram shows the common and unique OTUs in ileum samples in SS0.45 and SY0.45 groups. (H) PLS-DA plot of ileum microbiota between the SS0.45 and SY0.45 groups. (I) Relative abundance of the genera detected in the ileum in SS0.45 and SY0.45 groups. (J) Prediction of changed Kyoto Encyclopedia of Genes and Genomes (KEGG) pathways using Phylogenetic Investigation of Communities by Reconstruction of Unobserved States (PICRUSt) analysis in the SY groups compared with the SD group. (K) Prediction of changed KEGG pathways using PICRUSt analysis in the SS group compared with the SD group. (L) Prediction of changed KEGG pathways using PICRUSt analysis in the SY0.45 group compared with the SS0.45 group. (Bar plots on the left side displayed the mean proportion of each KEGG pathway. Dot plots on the right show the differences in mean proportions between the 2 indicated groups using *P*-values). SS = sodium selenite.

It is known that dietary Se supplementation can affect the composition of the gut microbiota, and the gut microbiota also influences Se bioavailability and selenoprotein expression in mice (Hrdina et al., 2009). Therefore, to assess the changes of microbiota in aged laying hens after Se supplementation, the 16S rRNA sequencing was performed. The sequence and OTUs data are shown in Fig. S2 and Fig. 4A. Compared with the SD group, SY supplementation had no significant effect on the α-diversity of the bacteria community (Fig. S2B). Partial Least Squares Discrimination Analysis (PLS-DA) showed there were significant differences in the composition of the microbiota (Anosim, *P* = 0.002) (Fig. 4B). At the phylum level, *Firmicutes* was dominated in 4 groups (Fig. S2C) and there were no significant changes in the abundance of bacteria (Table S4). In the genus level, the abundance of several bacteria such as *Veillonella* (*P* < 0.05) and *Campylobacter* (*P* < 0.05) was increased in SY groups, and SY supplementation markedly reduced the abundance of both *Stenotrophomonas* (*P* < 0.01) and *Faecalicoccus* (*P* < 0.05) (Fig. 4C).

Additionally, the results of inorganic selenium supplementation on ileum microbiota showed that there were no significant differences in the richness and diversity (Fig. S2E), but the microbial community was considerably different (Anosim, *P* = 0.049, *r* = 0.2216) (Fig. 4D and E). *Firmicutes* was dominated in 2 groups (Fig. S2F). Compared with the SD group, the abundance of *Lactobacillus* (*P* < 0.01) and *Stenotrophomonas* (*P* < 0.05) was noticeably reduced by sodium selenite supplementation, but *Romboutsia* (*P* < 0.01), *Turicibacter* (*P* < 0.05), *Veillonella* (*P* < 0.05), and *Corynebacterium_1* (*P* < 0.05) was dramatically increased in the genus level (Fig. 4F).

Compared to the SS0.45 group, there was no significant effect on the Chao1, Good coverage, observed species, and PD index of the bacteria community in the SY0.45 group. However, the Shannon and Simpson index were significantly higher in the SS0.45 group (Fig. S2H). There were statistical differences in the compositions of gut microbiota between the 2 groups (Anosim, *P* = 0.001, *r* = 0.5296) (Fig. 4G and H). The level of the phylum *Cyanobacteria* (*P* < 0.05) was significantly decreased in the SY0.45 group (Table S4). In the genus level, the relative abundance of *Romboutsia* (*P* < 0.01) and *Methylobacterium* (*P* < 0.05) was markedly reduced in the SY0.45 group, but *Lactobacillus* (*P* < 0.01) and *Escherichia-Shigella* (*P* < 0.05) were dramatically elevated (Fig. 4I).

To further understand the function of the altered gut microbiota by Se supplementation, the PICRUSt analysis was completed. Some enriched pathways, including nitrogen metabolism, MAPK signaling pathway-yeast and translation proteins of gut microbiota, were activated by both SY and sodium selenite supplementation, but the Pentose phosphate pathway was suppressed, compared to the SD group (Fig. 4J, K and Fig. S3). Additionally, SY supplementation might restrain steroid biosynthesis and metabolism, but sodium selenite supplementation might significantly suppress carbohydrate metabolism, lipid metabolism and other amino acids metabolism, it also might increase energy metabolism, amino acid metabolism and bacterial movement (Fig. 4J, K and Fig. S3). Moreover, the differences between SY and sodium selenite were majorly enriched in carbohydrate metabolism, lipid metabolism, amino acid metabolism, cell cycle as well as other biological processes (Fig. 4L and Fig. S4).

### 3.5 Correlation of transcriptome and microbiota reveals the effects of selenium yeast supplementation on laying rate and Se deposition in the ileum

**Fig. 5:**
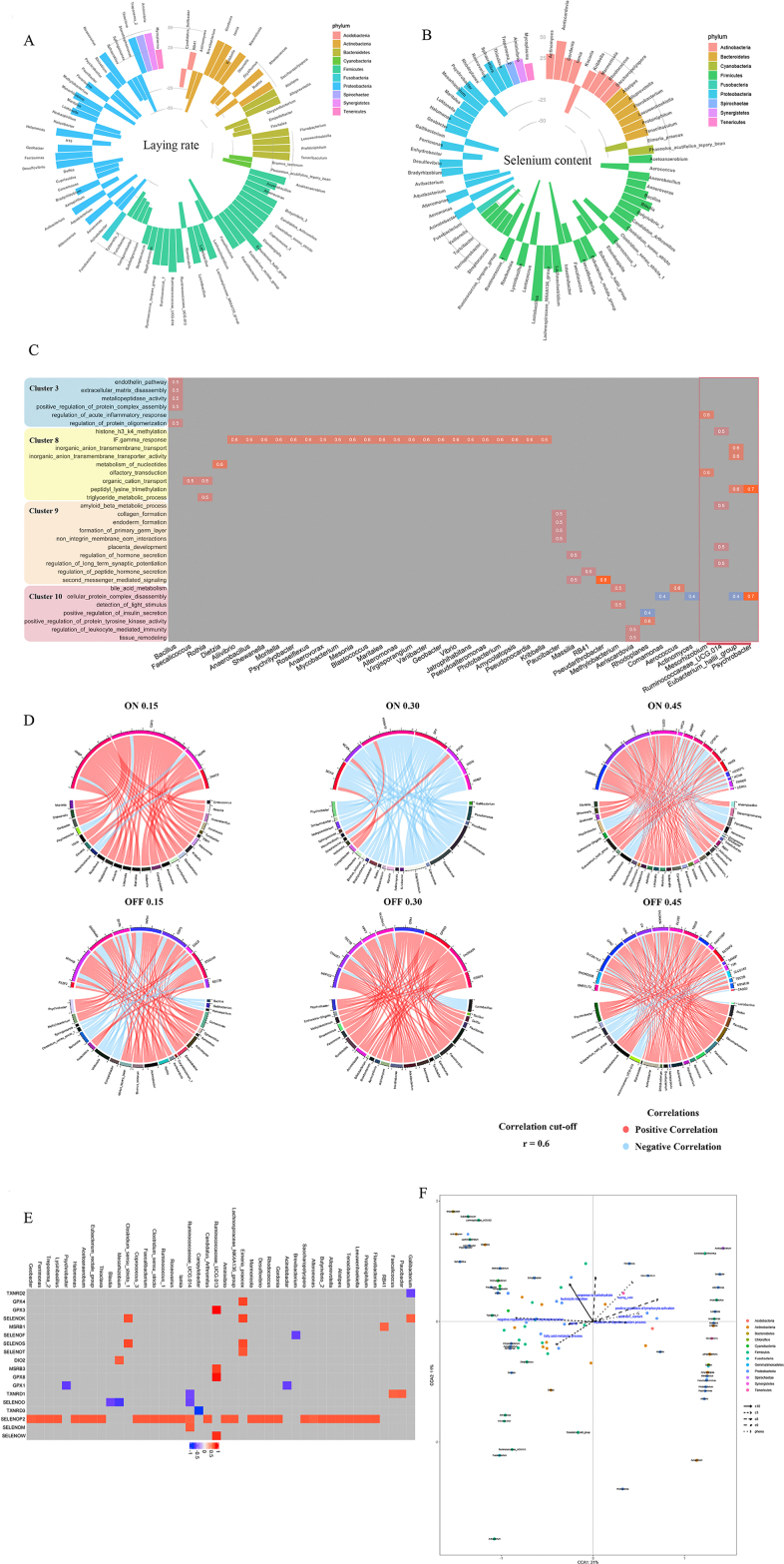
Associations between host transcriptome, microbiota, laying rate and selenium status during selenium yeast (SY) supplementation. (A) Circles plots display the correlation between the abundance of bacterial genera and laying rate. Rectangle towards outermost represents the relationship is positive, rectangle towards innermost represents the relationship is negative. (B) Circles plots display the correlation between the abundance of bacterial genera and selenium content in the ileum. Rectangle towards outermost represents the relationship is positive, rectangle towards innermost represents the relationship is negative. (C) A heatmap of significant correlations between pathways in specific clusters and genera abundance. (D) Circos plots of Pearson correlation analysis between the abundance of bacterial genera and the expression of ON as well as OFF gene in the ileum. Positive and negative correlations (*r* > 0.6) are displayed by red and blue links, respectively. (E) A heatmap of Pearson correlation analysis between the abundance of bacterial genera and selenoprotein gene expression. (F) Constrained correspondence analysis reveals the correlations among the relative abundance of the specific microbes, the selenium content in the ileum, laying rate, and the pathways in specific clusters.

To investigate the relationship between changes of host gene expression and ileum microbiota with laying rate and ileum Se content after SY supplementation, the following analysis was carried out. Pearson correlation analysis showed ileum Se content was positively correlated with *Mesorhizobium*, *Anaerobacillus* and *Eubacterium_hallii_group*, but negatively correlated with *Brevibacterium*, *Faecalicoccus* and *Stenotrophomonas* (Fig. 5A). Moreover, the laying rate was positively associated with *Lactobacillus*, *Anaerovorax* and *Geobacter*, but negatively associated with *Romboutsia*, *Turicibacter* and *Alteromonas* (Fig. 5B).

To further assess the relationship between microbiota and transcriptome, Pearson correlation analysis was performed. The abundance of *Mesorhizobium* and *Bacillus* was highly correlated with several pathways (regulation of acute inflammatory response, metallopeptidase activity and regulation of protein oligomerization, etc.) in cluster 3. Meanwhile, interferon-gamma response pathway of cluster 8 was highly associated with the abundance of *Anaerobacillus*, *Psychrilyobacter* and *Mycobacterium*, etc. The abundance of *Paucibacter* was hugely correlated with the pathways in cluster 9. Meanwhile, the abundance of *Methylobacterium*, *Aeriscardovia* and *Aerococcus* was greatly associated with the pathways of cluster 10 (Fig. 5C).

To elucidate the relationship between bacterial abundance and switch-on or switch-off genes, the Pearson correlation coefficient was calculated (Fig. 5D and Table S5). The abundance of *Stenotrophomonas*, *Acinetobacter* and *Psychrobacter* was negatively correlated with the expression of ON genes. However, the abundance of *Campylobacter*, *RB41* and *Eubacterium_hallii_group* was positively correlated with the expression of ON genes. Furthermore, the abundance of *Comamonas, Psychrobacter*, *Stenotrophomonas* and *Faecalicoccus* was positively correlated with the expression of OFF genes. Alternatively, the abundance of *Eubacterium_hallii_group*, *Campylobacter* and *RB41* was negatively correlated with the expression of OFF genes. In brief, SY supplementation directly influenced the biosynthesis of the selenoprotein. To gain further insight into the relationship between selenoprotein expression and microbiota, the Pearson correlation coefficient analysis (*P* < 0.01; *r* > 0.6) was conducted (Fig. 5E). *SELENOP2* had positive correlations with almost the whole microbiota, and *TXNRD3* was negatively correlated with *Campylobacter*. On the other hand, the expression of *GPX3*, *MSRB3*, *GPX8*, and *SELENOW* was positively associated with the abundance of *Ruminococcaceae_UCG.013*.

To provide an initial visualization of the relationships among Se content in the ileum, laying rate, transcriptome pathways of interest and microbiota CCA analysis was carried out (Fig. 5F). The results revealed that the ileum Se content was positively correlated with the positive regulation of lymphocyte activation (cluster 9), regulation of digestive system progress (cluster 8) as well as the abundance of *Anaerobacillus*, *Alteromonas*, and *Loktanella*. In contrast, aging (cluster 8), fatty acid metabolic process (cluster 3), and the abundance of *Streptococcus*, *Brevibacterium*, *Aerococcus*, and *Devosia* was inversely correlated with Se content. Moreover, the laying rate had a positive correlation with positive regulation of the lymphocyte activation (cluster 9), the response to carbohydrate (cluster 9), and the abundance of *Mesorhizobium* and *Paracoccus*, but had a negative relationship with fatty acid metabolic progress (cluster 3), the abundance of *Streptococcus*, *Devosia*, *Aerococcus*, and *Intestinibacter*.

## 4 Discussion

The poultry industry aims to improve productivity and maintain the health as well as the welfare of flocks (Gangadoo et al., 2018). Several studies suggested that intestinal health and microbiota status was closely associated with the productivity of hens because the intestine played a critical role in nutrient digestion and absorption (Wang et al., 2020). In this study, 0.30 mg/kg SY supplementation for 12 weeks significantly alleviated the decline of laying rate, similar to what has been previously found in aged broiler breeder hens (Emamverdi et al., 2019). Therefore, it is inferred that supplementing SY may improve the laying production in aged laying hens. Moreover, some significant genes and microbiota related to egg production were identified. *PSCA* was identified as the “ON gene” in 0.30 mg/kg Se supplementation, possibly played a special role in follicle selection which was an important process affecting laying performance (L Chen et al., 2020). In addition, the results showed that *PSCA* had a positive relationship with the abundance of propionate producer *Veillonella*, and a negative relationship with opportunistic pathogens *Stenotrophomonas*. Meanwhile, a significant positive correlation between the abundance of *Lactobacillus* and the laying rate was obtained. Similar results had proved in a previous study that the abundance of *Lactobacillus* was higher in the high egg-laying performance group in comparison with the low egg-laying performance group (Elokil et al., 2020). A 0.6% *Lactobacillus* supplement in the diet increased the egg production of feeding laying hens (Choe et al., 2012). Therefore, the laying rate was significantly higher in the SY0.30 group, which may be due to the switching on *PSCA* and the increasing beneficial bacteria *Lactobacillus*.

The transcriptome results revealed that SY supplementation changed the expression of genes associated with Glycerophospholipid and Glycerolipid metabolism in the ileum. This result was consistent with the previous studies that showed differential expressed genes (DEG) were mainly enriched in Glycerolipid metabolism after 200 μmol/L Se treatment in silkworm (Jiang et al., 2020), suggesting that Se supplementation has a potential effect on Glycerolipid metabolism in livestock and poultry. Similar results were confirmed in WGCNA analysis, the genes in modules that were significantly associated with ileum Se content were enriched in fatty acid metabolism. Coherently with previous studies, selenium supplementation tended to activate fatty acid metabolism pathways in mice (Hu et al., 2018). In addition, several candidate hub genes related to Se deposition were identified. *TXNRD1* played an important role in modulating redox signaling and maintaining intestinal health (Li et al., 2019). *DLD* is a key enzyme involved in energy metabolism (Yang et al., 2007) and Citrate cycle/TCA cycle, which provides the intermediates for the metabolism of amino acids, nucleic acids, carbohydrates and lipids. *ILK* has a key role in the regulation of cell migration, and the knockdown of *ILK* in human intestinal cells severely inhibited its spreading and migration (Yuan et al., 2011). These results suggest that SY supplementation (discarding dose effect) primarily influence lipid metabolism in the ileum.

After SY treatment, the expression of selenoprotein genes in the ileum clustered into 3 patterns. *GPX4*, *MSRB1*, *SELENOF*, *SELENOT*, *SELENOK*, *SELENOS* were clustered into a similar expression pattern. It was proved that *GPX4* played an important role in oxidative phosphorylation as well as mitochondrial dysfunction pathways to protect mitochondria from oxidative damage (Cole-Ezea et al., 2012). *MSRB1* (Lee et al., 2017), *SELENOK* (Nettleford and Prabhu, 2018) and *SELENOS* (Speckmann et al., 2014) were involved in inflammatory responses and intestinal health. The expression of *DIO2*, *GPX1*, *GPX3*, *GPX7*, *GPX8*, *MSRB3*, *SELENOP2*, *SELENOM*, and *SELENOW* increased accordingly to the rise of selenium dose. *GPX1* and *GPX3* are the major components in the Glutathione peroxidase family, they are responsible reduce hydrogen peroxide, organic hydroperoxides and/or phospholipid hydroperoxides with a vital role in the amelioration of peroxide-mediated deleterious effects (Peters et al., 2018). Moreover, *SELENOW* and *SELENOT* have antioxidant functions (Dikiy et al., 2007) and *SELENOM* encodes oxidoreductase (Labunskyy et al., 2007). These selenoproteins were regarded as direct or indirect potential regulators of oxidative/redox balance (Li et al., 2018) modulating intestinal health. Indeed, most of these selenoproteins (*GPX1*, *GPX3*, *GPX4*, *SELENOM, SELENOU*) are responsive to alterations of Se status. Thus, we speculated that SY supplementation can affect the health and functions of the intestines by regulating selenoprotein expression. The other 2 selenoproteins, namely *TXNRD2* and *TXNRD3,* could maintain proliferation and differentiation processes by regulating the Wnt pathway (Kipp et al., 2012). Consistently with other studies, the expressions of *TXNRD2* and *TXNRD3* were the highest in the selenium deficiency group (Kipp et al., 2009). These results suggested that SY regulated the expressions of selenoprotein genes to affect the health and function of intestines through regulating oxidative/redox balance, maintaining proliferation and differentiation processes as well as metabolism balance.

Moreover, significant increasing abundance of *Veillonella* and decreasing abundance of *Stenotrophomonas* were observed after SY supplementation, which might benefit chicken's intestinal health. Trials on both animals and humans have confirmed that *Veillonella* was a producer of short-chain fatty acid acetate and propionate, and maintained energy homeostasis (Ríos-Covián et al., 2016), suggesting that increasing the abundance of this family might benefit poultry health. *Stenotrophomonas* is considered as an opportunistic pathogen (Maes et al., 2019) and can consequently be a potential risk for animal health. It was reported that SY interfered with the diversity of gut microbiota and reduced the abundance of harmful bacteria caused by Ochratoxin-A (Yang et al., 2020). Based on PICRUSt analysis results, it is inferred that SY may enhance the energy metabolism of gut microbiota by increasing the abundance of *Veillonella*.

Based on functional enrichment by transcriptome and PICRUSt analysis, the common differential pathways obtained were Glycerolipid metabolism, Glycerophospholipid metabolism, ABC transporters, as well as MAPK signaling pathway, suggesting that the differential effects between SY and sodium selenite on eukaryotic and prokaryotic organisms were conservative. Differences of genera microbiota between the SY and SS group were mainly in *Lactobacillus*, *Escherichia-Shigella*, etc. The increased abundance of the *Lactobacillus* demonstrated that SY supplementation in aged laying hens might contribute to improving gut health, similar to what has been previously found in the 0.9 mg/kg selenium nanoparticles supplementation trial (Yang et al., 2020).

Detailed information indicated that the gut microbiota was related to plasma levels of Se in mice (Hrdina et al., 2009). In this study, *Eubacterium_hallii_group* as a member of the butyrate-producers (Q Chen et al., 2020) had a positive relationship with Se content, whereas the *Stenotrophomonas* − an opportunistic pathogen − had a negative relationship with Se content. These 2 results suggested that Se status in the ileum of aged laying hens may modulate the balance of the beneficial bacterium and pathogens. Correlational analyses showed that the pathways in cluster 3 were significantly related to *Bacillus*. *Bacillus* as probiotics that could enhance gut health (Zhang et al., 2021) and produce a variety of enzymes such as protease, amylase and lipase (Bai et al., 2018). Meanwhile, IFN gamma response in cluster 8 was significantly correlated with most of the microbiota, highlighting that SY might modulate the interactions of immune response and microbiota in the ileum. Interestingly, *Ruminococcaceae_UCG.014* was remarkably associated with the pathways in 4 clusters, which affected the maintenance of gut health and had the enzymatic ability to degrade cellulose and hemicellulose (Dai et al., 2018). These results indicated that the changes in pathways and microbiota affected by Se supplementation were a major factor involved in immune response and metabolism, which is similar to the functional enrichment analysis results.

CCA analysis results showed that Se content was positively correlated with some pathways and bacteria which are associated with immune response and digestive system progress, it has been reported that *Loktanella* was enriched in diseased tissues, suggesting it could be a candidate opportunistic pathogen (Zhang et al., 2020). *Anaerobacillus* grown under alkaliphilic or halophilic conditions through fermentative or anaerobic respiration (Bassil and Lloyd, 2017) might take part in the regulation of digestive system progress and intestinal environment. These results suggested that SY supplementation might balance the special bacteria by activating immune response and digestive progress to improve the ileum environment.

## 5 Conclusions

**Fig. 6:**
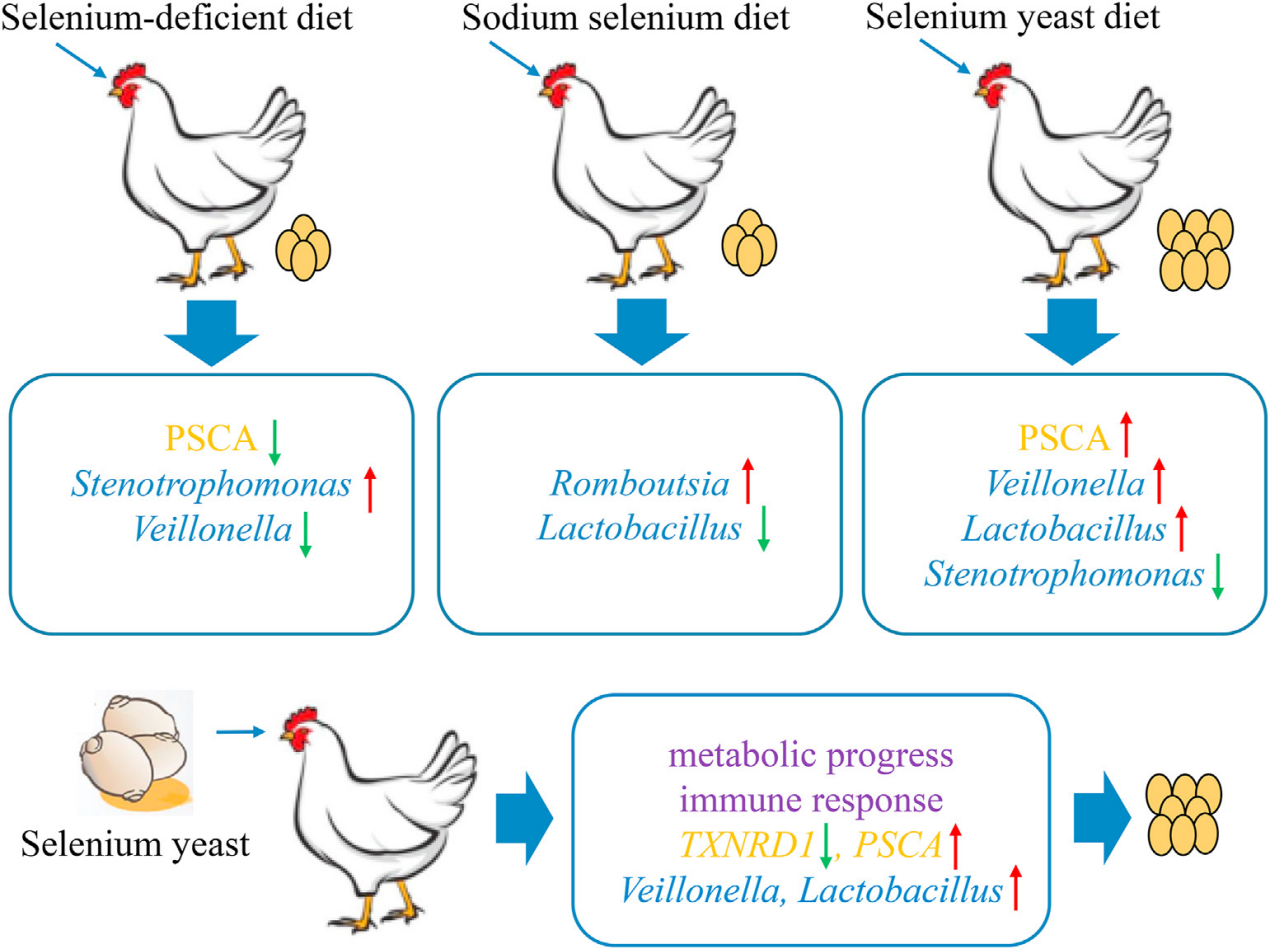
The schematic map of the relationship among selenium yeast (SY), laying performance, ileum microbiota and gene expression in aged laying hens. *PSCA* = Prostate stem cell antigen; *TXNRD1* = Thioredoxin reductase 1.

In summary, our results revealed that 0.30 mg/kg SY supplementation could slow down the deterioration in egg production in aged laying hens. In addition, the transcriptome functional enrichment analysis showed that SY supplementation might affect the metabolic processes (Glycerolipid and Glycerophospholipid metabolism), the immune response as well as intestinal absorption. Furthermore, 16S rRNA analysis suggested that Se supplementation could modulate the composition of gut microbiota by increasing the abundance of specific beneficial bacteria including *Veillonella*, *Turicibacter*, *Lactobacillus*, but decreasing the abundance of pathogenic bacteria *Stenotrophomonas*. Further, integrated analysis and CCA analysis suggested that the changes of pathways and microbiota affected by Se were major factors involved in metabolism and immune response, and explained the relationship between diet, host gene expression, microbiota and laying performance (Fig. 6). From these relationships, we inferred that changes in ileum affected by Se may have a profound effect on egg production in aged laying hens.

## Author contributions

**Zhexi Liu** carried out the animal experiments, performed sample analysis and wrote the manuscript. **Yutao Cao** performed bioinformatics data analysis. **Yue Ai** took part in animal feeding and assisted with sample collection. **Xiaonan Yin** provided the selenium resource. **Linli Wang** and **Mengyao Wang** assisted with sample collection. **Bingkun Zhang** and **Yuming Guo** designed experiments and contributed to developing conceptual ideas. **Zhengxing Lian** and **Keliang Wu** contributed to developing conceptual ideas. **Hongbing Han** designed experiments, interpreted the results and revised the original draft of the manuscript.

## Declaration of competing interest

We declare that we have no financial and personal relationships with other people or organizations that can inappropriately influence our work, and there is no professional or other personal interest of any nature or kind in any product, service and/or company that could be construed as influencing the content of this paper.
