# Supplementary material for: Microbiome and ileum transcriptome revealed the boosting effects of selenium yeast on egg production in aged laying hens
Source: Anim Nutr. 2022 Apr 21;10:124–36. doi: 10.1016/j.aninu.2022.04.001 (PMC9136271; doi:10.1016/j.aninu.2022.04.001)
Supplement: Multimedia component 1 [file mmc1.pdf]

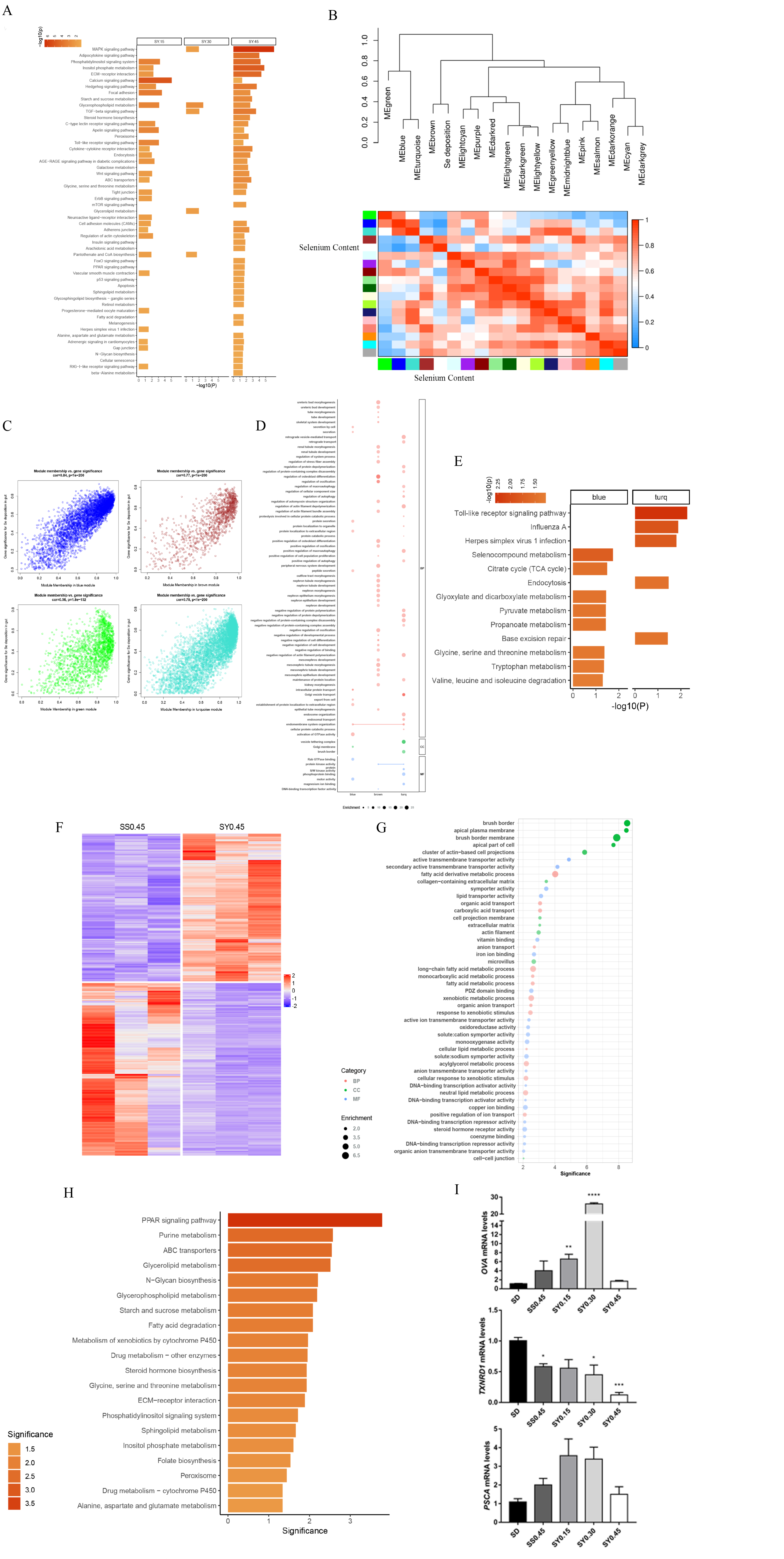

Fig. S1 . Transcriptome supplementary information.

(A) KEGG enrichment analysis of DEG was identified between pairs of conditions (SD vs SY0.15, SD vs SY0.30, and SD vs SY0.45).

(B) Relationships between modules. The top part is the dendrogram of module eigengenes. The bottom part is a heatmap plot of the adjacencies of modules.

(C) Gene significance (y - axis) vs intramodular connectivity (x - axis), plotted separately for 4 selected modules in different sequencing data sets.

(D) Enriched GO categories for genes identified among 3 modules (brown, blue, and turquoise modules) genes.

(E) KEGG enrichment analysis of genes in modules (blue and turquoise modules).

(F) Hierarchical clustering of DEG between the SS0.45 group and SY0.45 group.

(G) Enriched GO categories for DEG identified in SS0.45 group and SY0.45 group, determined using the Gene list analysis tool on PANTHER website.

(H) KEGG enrichment analysis of DEG between the SS0.45 group and SY0.45 group.

(I) Validation of the DEG and hub genes from the transcriptomic analysis. * means $P < 0.05$, ** means $P < 0.01$, compared to the SD group.
