# Supplementary material for: Microbiome and ileum transcriptome revealed the boosting effects of selenium yeast on egg production in aged laying hens
Source: Anim Nutr. 2022 Apr 21;10:124–36. doi: 10.1016/j.aninu.2022.04.001 (PMC9136271; doi:10.1016/j.aninu.2022.04.001)
Supplement: Multimedia component 2 [file mmc2.pdf]

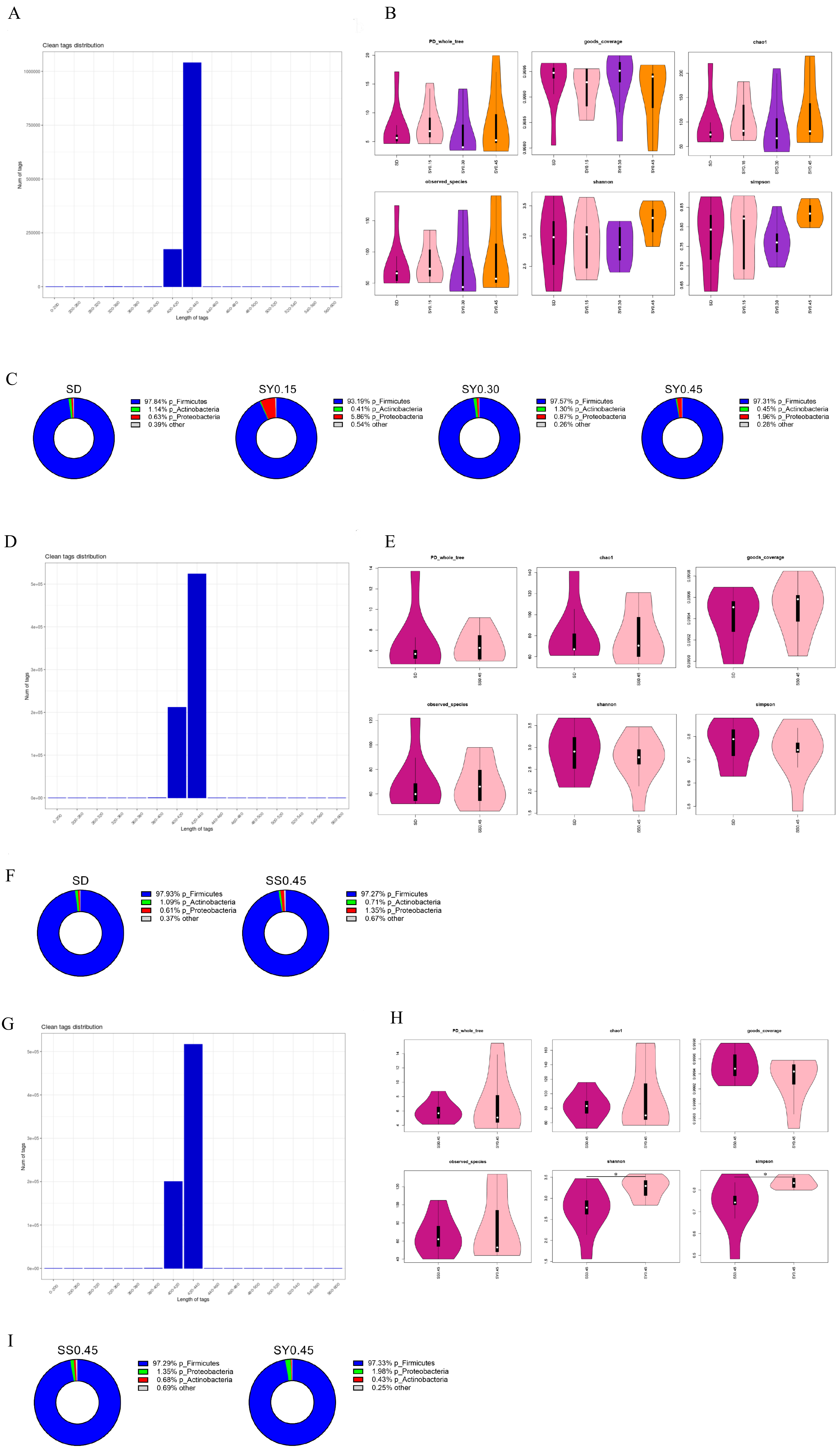

Fig. S2. Basic 16S rRNA analysis including lengths of tags, alpha-diversity and relative phylum abundances.
(A) Quality sequence distribution statistics between the SD group and SY groups from 16S rRNA sequencing.
(B) Violin plot of alpha diversity index values between the SD group and SY groups from 16S rRNA sequencing.
(C) Relative abundance of the gut microbiota at the phylum level from the SD group and SY groups.
(D) Quality sequence distribution statistics between the SD group and SS group from 16S rRNA sequencing.
(E) Violin plot of alpha diversity index values between the SD group and SS group from 16S rRNA sequencing.
(F) Relative abundance of the gut microbiota at the phylum level from the SD group and SS group.
(G) Quality sequence distribution statistics between the SS0.45 group and SY0.45 group from 16S rRNA sequencing.
(H) Violin plot of alpha diversity index values between the SS0.45 group and SY0.45 group from 16S rRNA sequencing.
(I) Relative abundance of the gut microbiota at the phylum level from the SS0.45 group and SY0.45 group.
