# Supplementary figures and images for: Microbiome and ileum transcriptome revealed the boosting effects of selenium yeast on egg production in aged laying hens
Source: Anim Nutr. 2022 Apr 21;10:124–36. doi: 10.1016/j.aninu.2022.04.001 (PMC9136271; doi:10.1016/j.aninu.2022.04.001)

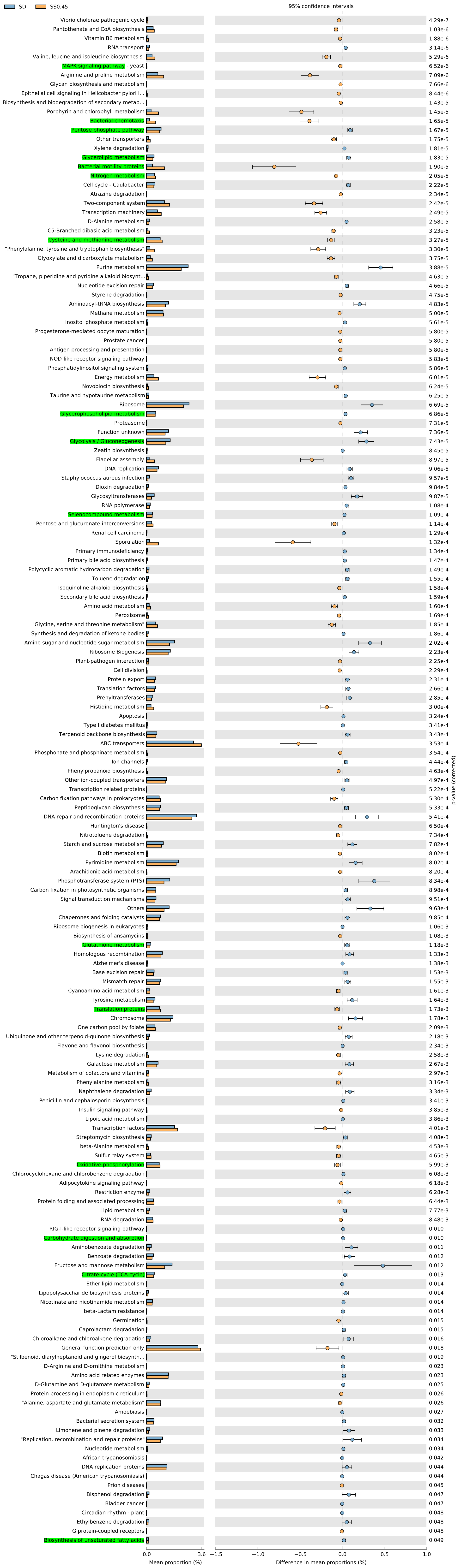

Supplement: Multimedia component 3 [file mmc3.pdf]

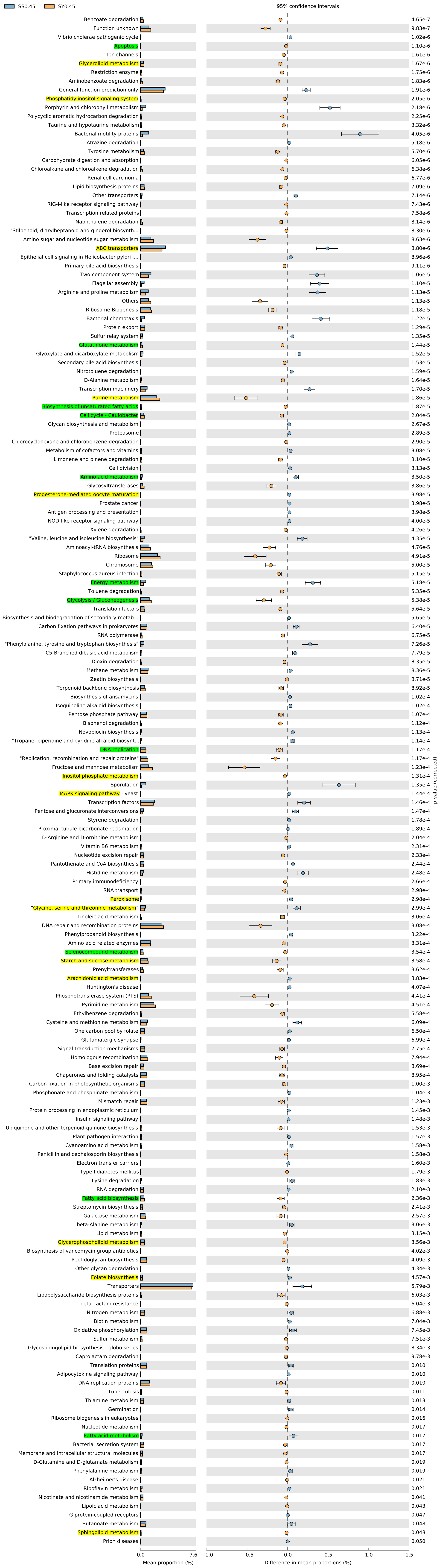

Supplement: Multimedia component 4 [file mmc4.pdf]
